# Successful double valve replacement with extensive annular enlargement using the Commando procedure in a patient with small aortic and mitral annuli after previous double valve surgery

**DOI:** 10.1093/jscr/rjaf1025

**Published:** 2026-01-02

**Authors:** Akito Inoue, Daisuke Takeyoshi, Hidenobu Akamatsu, Tasuku Kawarabayashi, Jeonga Lee, Jun Maruoka, Kentaro Shirakura, Yuki Setogawa, Ryo Okubo, Hiroyuki Miyamoto, Ryohei Ushioda, Aina Hirofuji, Shogo Takahashi, Shingo Kunioka, Hiroyuki Kamiya

**Affiliations:** Department of Cardiac Surgery, Asahikawa Medical University, Midorigaoka 1-1-1, Asahikawa, Hokkaido 078-8510, Japan; Department of Cardiac Surgery, Asahikawa Medical University, Midorigaoka 1-1-1, Asahikawa, Hokkaido 078-8510, Japan; Department of Cardiac Surgery, Asahikawa Medical University, Midorigaoka 1-1-1, Asahikawa, Hokkaido 078-8510, Japan; Department of Cardiac Surgery, Asahikawa Medical University, Midorigaoka 1-1-1, Asahikawa, Hokkaido 078-8510, Japan; Department of Cardiac Surgery, Asahikawa Medical University, Midorigaoka 1-1-1, Asahikawa, Hokkaido 078-8510, Japan; Department of Cardiac Surgery, Asahikawa Medical University, Midorigaoka 1-1-1, Asahikawa, Hokkaido 078-8510, Japan; Department of Cardiac Surgery, Asahikawa Medical University, Midorigaoka 1-1-1, Asahikawa, Hokkaido 078-8510, Japan; Department of Cardiac Surgery, Asahikawa Medical University, Midorigaoka 1-1-1, Asahikawa, Hokkaido 078-8510, Japan; Department of Cardiac Surgery, Asahikawa Medical University, Midorigaoka 1-1-1, Asahikawa, Hokkaido 078-8510, Japan; Department of Cardiac Surgery, Asahikawa Medical University, Midorigaoka 1-1-1, Asahikawa, Hokkaido 078-8510, Japan; Department of Cardiac Surgery, Asahikawa Medical University, Midorigaoka 1-1-1, Asahikawa, Hokkaido 078-8510, Japan; Department of Cardiac Surgery, Asahikawa Medical University, Midorigaoka 1-1-1, Asahikawa, Hokkaido 078-8510, Japan; Department of Cardiac Surgery, Asahikawa Medical University, Midorigaoka 1-1-1, Asahikawa, Hokkaido 078-8510, Japan; Department of Cardiac Surgery, Asahikawa Medical University, Midorigaoka 1-1-1, Asahikawa, Hokkaido 078-8510, Japan; Department of Cardiac Surgery, Asahikawa Medical University, Midorigaoka 1-1-1, Asahikawa, Hokkaido 078-8510, Japan

**Keywords:** commando procedure, double valve replacement, redo cardiac surgery, small aortic annulus, patient–prosthesis mismatch

## Abstract

We report a 70-year-old woman (body surface area 1.42 m^2^) undergoing redo double valve replacement with extensive annular enlargement using the Commando procedure. Thirteen years earlier, she received aortic valve replacement (St. Jude Medical 19 mm), mitral repair (Physio Ring 24 mm), and tricuspid annuloplasty. She presented with progressive heart failure; echocardiography showed severe mitral stenosis (mitral valve area 0.96 cm^2^) and moderate aortic stenosis with moderate paravalvular leak. To avert patient–prosthesis mismatch (PPM), both annuli were enlarged with bovine pericardium, permitting implantation of an Epic 27 mm mitral and an Inspiris 23 mm aortic bioprosthesis. Postoperative echocardiography demonstrated excellent hemodynamics (aortic effective orifice area 1.76 cm^2^, mean gradient 6 mmHg; mitral mean gradient 3 mmHg). Recovery was uneventful, and she remains asymptomatic. Commando surgery enabled safe, simultaneous enlargement of both annuli, minimizing PPM, and preserving options for future valve-in-valve therapy.

## Introduction

The Commando procedure enables simultaneous reconstruction of the aortic and mitral annuli and the aorto-mitral fibrous continuity. Initially developed for invasive endocarditis, it has become an option in reoperations requiring complex annular enlargement. Redo double valve replacement (DVR) with small annuli presents particular challenges: patient–prosthesis mismatch (PPM) is frequent, and valve-in-valve (ViV) transcatheter therapy may be compromised. We report a patient who underwent redo DVR with enlargement of both aortic and mitral annuli using the Commando procedure to avoid PPM and secure the potential for future ViV therapy.

## Case report

**Figure 1:**
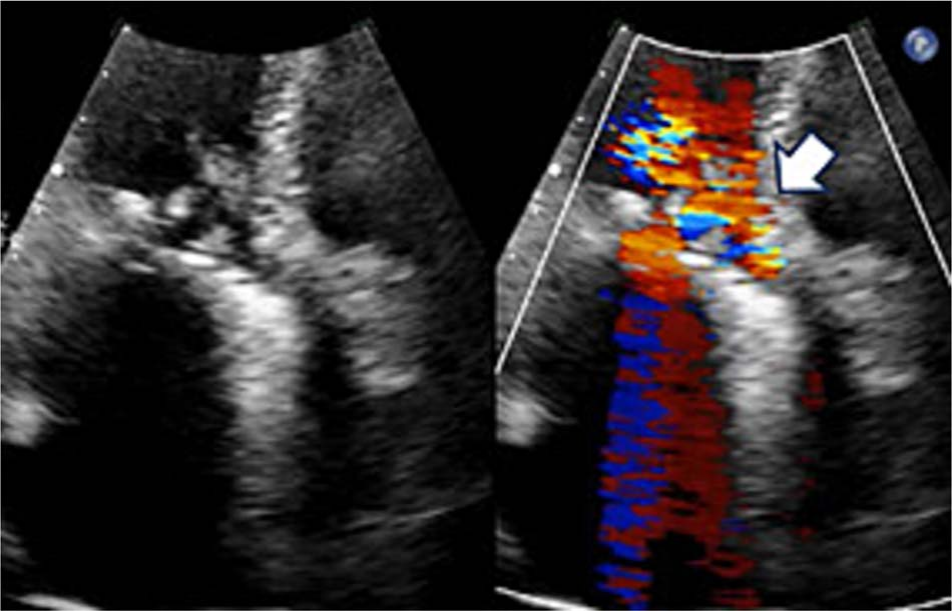
Preoperative transthoracic echocardiography: significant paraventricular leakage was shown with the white arrow.

A 70-year-old woman (body surface area [BSA] 1.42 m^2^) had undergone aortic valve replacement (St. Jude Medical 19 mm mechanical aortic valve; Abbott, St. Paul, MN, USA), mitral valve repair (Physio Ring 24 mm; Edwards Lifesciences, Irvine, CA, USA), and tricuspid annuloplasty with De Vega technique 13 years earlier. Over the previous two years, she experienced progressive heart failure despite optimized medical therapy. Echocardiography demonstrated severe mitral stenosis with a mitral valve area of 0.96 cm^2^, markedly restricted leaflets, and calcification of both commissures. The aortic prosthesis showed a peak velocity of 3.3 m/s with moderate stenosis and moderate paravalvular regurgitation (Fig. 1). Given persistent symptoms and limited efficacy of medical management, surgical intervention was planned. Her comorbidities included hypertension and chronic atrial fibrillation.

**Figure 2:**
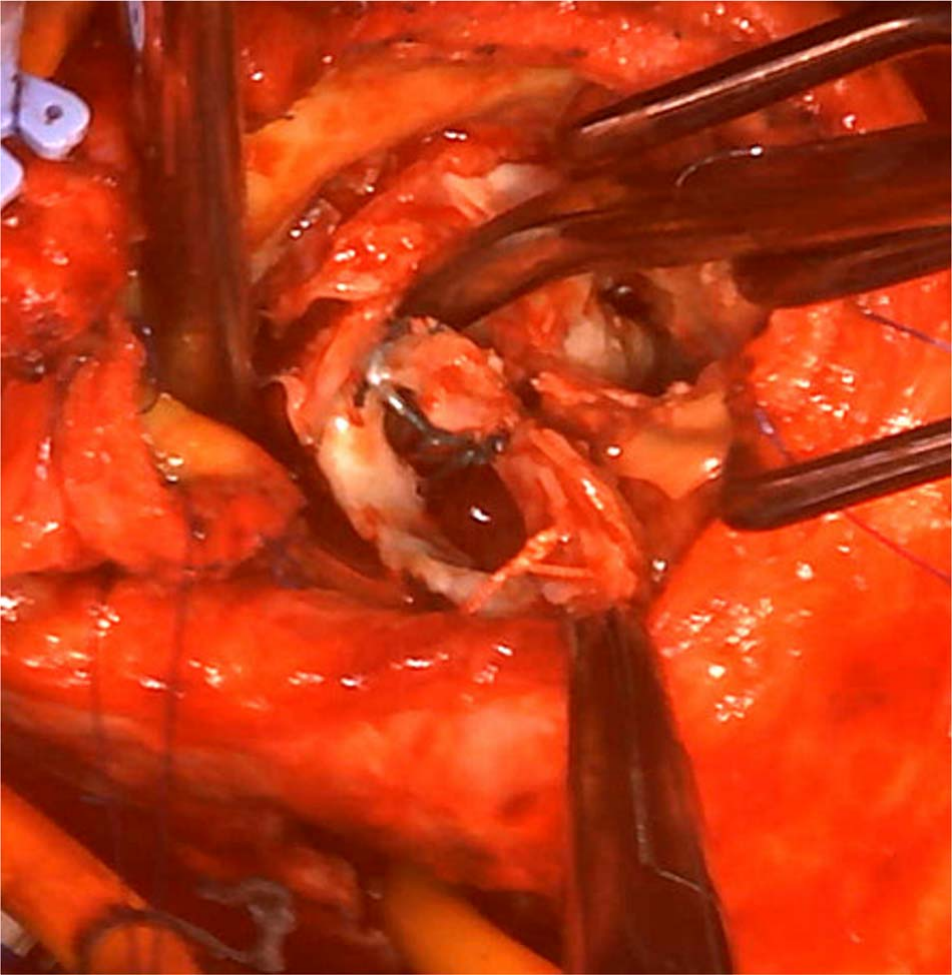
Intraoperative view: the aortotomy extended from the left-noncoronary commissure toward the left atrium.

The operation was performed through a redo median sternotomy. Cardiopulmonary bypass was established using ascending aortic cannulation and bicaval drainage. After cardioplegic arrest, the previous aortic prosthesis was explanted along the old aortotomy. The incision was then extended through the left-noncoronary commissure to open the left atrium (Fig. 2). The mitral annuloplasty ring and anterior leaflet were excised. Annular calcification was mild, and an edge-to-edge repair had been performed on the lateral side. The subvalvular tissue, including the lateral chordae, was shortened, whereas the anterior papillary muscle was elongated.

**Figure 3:**
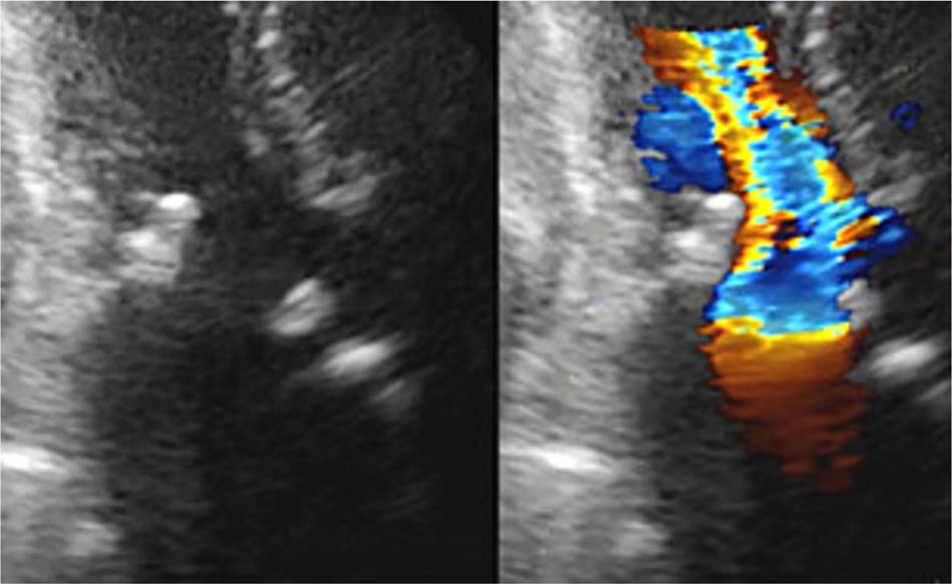
Postoperative transthoracic echocardiography: normal function of both prosthetic valves without dysfunction.

Seven pledgeted 2–0 Cardioxyl sutures were placed along the posterior mitral annulus, and a 27 mm Epic bioprosthesis (Abbott, St. Paul, MN, USA) was secured over two-thirds of the circumference. A folded 5 cm bovine pericardial patch (Edwards Lifesciences, Irvine, CA, USA) was attached to the remaining one-third using a 4–0 Prolene polypropylene running suture to create the left atrial roof and reconstruct the left ventricular outflow tract and noncoronary sinus of Valsalva. The aortic annulus was then reconstructed. The left and right coronary commissures were sutured from the left ventricular outflow tract toward the aortic side, while the noncoronary commissure was sutured from the external pericardial patch inward. A 23 mm Inspiris Resilia bioprosthesis (Edwards Lifesciences, Irvine, CA, USA) was implanted, and the remaining pericardium was used to close the aortotomy. The patient was extubated on postoperative Day 1 but required reintubation the same day because of pneumonia, which responded to antibiotics, allowing re-extubation on Day 7. Postoperative echocardiography revealed excellent hemodynamics: aortic valve effective orifice area (EOA) 1.76 cm^2^ with peak velocity 1.8 m/s and mean gradient 6 mmHg; mitral valve peak velocity 1.9 m/s and mean gradient 3 mmHg (Fig. 3). She was discharged on Day 21 and has remained free of heart failure.

## Discussion

Redo DVR in patients with small aortic and mitral annuli remains technically demanding. Concomitant enlargement of both annuli is often essential to implant adequately sized prostheses and prevent PPM, a well-recognized predictor of adverse outcomes, including persistent symptoms, higher gradients, and reduced long-term survival. In our patient, the prior 19 mm St. Jude Medical mechanical aortic valve yielded an estimated indexed EOA (iEOA) of 0.83 cm^2^/m^2^, already in the moderate PPM range. Manufacturer data for the Inspiris bioprosthesis indicate predicted iEOAs of 0.72 cm^2^/m^2^ for 19 mm, 0.84 cm^2^/m^2^ for 21 mm, and 1.00 cm^2^/m^2^ for 23 mm valves, underscoring that at least a 23 mm prosthesis was needed to avoid PPM [1]. Given the markedly reduced mitral valve area of 0.96 cm^2^ and the presence of a rigid annuloplasty ring, the annular exposure and flexibility were expected to be restricted, making it difficult to implant a prosthesis larger than ~25 mm and to achieve an optimal postoperative gradient.

The Commando procedure provided a comprehensive solution. By incising through the left-noncoronary commissure and using a large pericardial patch, we created generous exposure and achieved simultaneous enlargement of both annuli. This allowed implantation of an Inspiris 23 mm aortic valve and an Epic 27 mm mitral valve, increasing the iEOA of the aortic prosthesis from 0.83 to 1.27 cm^2^/m^2^ and the mitral valve area from 0.96 to 2.32 cm^2^. These improvements ensured low transvalvular gradients and optimized hemodynamics.

Our experience aligns with previous reports supporting the efficacy of Commando enlargement in non-infective indications. Giambuzzi *et al*. [2] demonstrated that this approach enables the insertion of larger prostheses in patients with small annuli while maintaining acceptable mid- to long-term outcomes. Kinami *et al*. [2] showed significant improvements in Z-scores and mean pressure gradients after double annular enlargement in pediatric cases. Yang *et al*. [4] reported favorable early results with a “Chimney Commando” technique in redo DVR, confirming its utility in complex reoperations. The present case adds to this growing evidence by illustrating that Commando surgery can be applied safely to adult redo DVR for the dual goals of annular enlargement and long-term valve strategy.

Importantly, achieving large annuli also preserves the option of future ViV transcatheter therapy. As structural valve degeneration inevitably occurs, ViV procedures are increasingly utilized; their success depends on initial prosthesis size and the absence of significant PPM. By upsizing both valves in this case, we not only optimized immediate postoperative hemodynamics but also facilitated potential minimally invasive reintervention decades later.

In summary, the Commando procedure provided a comprehensive and durable solution for a complex redo DVR with small aortic and mitral annuli. By enabling concomitant enlargement of both annuli, it prevented PPM, ensured excellent early hemodynamics, and preserved the feasibility of future ViV therapy. This case highlights the expanding role of the Commando approach beyond infective endocarditis to include redo operation scenarios where maximal annular enlargement is mandatory for optimal patient outcomes.
